# Learning-by-Concordance for Family Physicians: Revealing its Value for Continuing Professional Development in Dermatology

**DOI:** 10.15694/mep.2018.0000236.1

**Published:** 2018-10-17

**Authors:** Julie Lecours, Fanny Bernier, Dominique Friedmann, Vincent Jobin, Bernard Charlin, Nicolas Fernandez

**Affiliations:** 1 Université de Montréal

**Keywords:** Learning-by-concordance, continuous professional development, dermatology, family physicians, work-based learning, distance education

## Abstract

This article was migrated. The article was marked as recommended.

Introduction

Continuous Professional Development (CPD) is an important part of a physician’s professional life. Yet, providing effective in-service training solutions is a persistent challenge for CPD planners.

Methods

Primary care physicians are frequently confronted with skin lesionsthey feel ill-prepared to manage. A dermatology Learning-by-concordance (LbC) online activity was developed and offered to family physicians for CPD credit. We were interested in finding out whether this online tool was suitable for CPD. Following a pilot phase, the on-line activity was launched and 45 geographically dispersed primary care physicians completed it. They participated in a telephone conference a week later with an expert to discuss outstanding questions. Evaluation was carried out by a survey that was available immediately after the last case.

Results

Participants found the on-line training tool user friendly and should be implemented on a larger scale. Participants found the dermatology concepts discussed allowed them to increase their knowledge and apply it to their practice.

Discussion

Among the strengths of LbC is that the learning task resemble those of a primary physician’s daily practice. Finally, our study reveals that LbC is easily integrated in busy work schedules and thus is an effective learning solution for CPD.

## Background

Continuous Professional Development (CPD) is an important part of a physician’s professional life. ( Mazmanian and Davis, 2002) A persistent challenge for CPD planners is providing accessible training for practicing clinicians juggling clinical work and personal life. ( Davis et al., 2013) Given budgetary constraints and increased patient case-loads, the traditional way of conducting CPD, based on face-to-face meetings and lectures given by experts, is being challenged. An innovative solution is required that affords the possibility of distributed specialized training to a geographically dispersed group of busy in-service professionals. This solution would provide access to expertise in a specific field, flexibility for course completion and minimum disruption of work schedules. ( Cabana et al., 2006; Brar et al., 2011; Batalden, Warm and Logio, 2013; Aldridge et al., 2015)

Primary care physicians are frequently confronted with skin lesions they feel ill-prepared to manage. ( Eichenfield et al., 2014) Primary care physicians recognized the need to brush up on their dermatology knowledge acquired during their initial training. The provincial association of family doctors approached the CPD team at the Faculty of medicine at the Université de Montréal, for training on this topic. Their members are dispersed across the province, often practice at sites that are far from university centers making it difficult to request help from dermatologists. The challenge of delivering this curriculum was resolved by resorting to Learning-by-concordance. This approach, based on script theory, ( Charlin, Tardif and Boshuizen, 2000; Lubarsky et al., 2015) is increasingly being used in undergraduate and graduate medical training and is now being adapted to CPD.

### The LbC Dermatology learning tool for primary care physicians

Learning-by-concordance (LbC) has been developed as an online platform that can disseminate a wide range of training contents. ( Foucault et al., 2015; Fernandez et al., 2016) The principal strength of LbC is that the clinical learning tasks resemble tasks of a physician’s daily practice. The learning model stems from cognitive apprenticeship theory ( Brown, Collins and Duguid, 1989; Shaddel et al., 2016) as participants can confront their answers to those of experts in the field and reflect upon the differences. Finally, the LbC online tool can be completed anytime and anywhere, thus resolving many constraints associated with traditional CPD activities.

Instead of delivering content material, as it is in text books, material in the LbC tool is embedded in clinical vignettes. The vignettes comprise four components: 1) a clinical situation described in a short statement or a photograph, along with the following prompt: “given the case above, your first thought is..”. Then, 2) a further piece of information is presented that might modify the first thought and, 3) participants must indicate to what extent the new information has modified their first thought. Once participants submit their response, they can access the fourth component: 4) physicians, considered experts in the specific field of medical practice, provide explanations and justifications for the answers they give (see Fernandez et al. for more details).

## Methods

The dermatology LbC online tool was developed by a team of Dermatologists in the residency program at the Université de Montréal. Primary care physicians from the province of Quebec were invited to take it as a CPD credited activity.

### Development of tool - Piloting phase

Based on dermatology referrals received at the Université de Montréal teaching hospital from family physicians province-wide, a senior resident (R-5) in dermatology reviewed the types of cutaneous lesions most frequently referred and organized them into 10 distinct groups - each corresponding to an eventual teaching module.

For the first module, on maculopapular rashes, 8 clinical vignettes were designed in accordance to learning objectives and LbC principles. The vignettes incorporated photographs about which 2 to 4 questions were asked. These questions mimicked the questions physicians would ask themselves upon seeing the lesions in a clinical context (diagnosis, investigation or treatment).

The pilot phase consisted of testing the 8 clinical vignettes with a small number of family physicians (N=5). In order to collect their comments and suggestions, a focus group was held with this pilot group and their comments and suggestions led to some adjustments for the final version of the training tool that was implemented. The entire module was designed on a Qualtrics Survey Software ( https://www.qualtrics.com/lp/survey-platform) platform for easy dissemination and response compilation.

### Implementing the dermatology LbC - DPC online tool

Participants could enroll for the training from the 18th of January to the 16th of March. The cost was 60$ CAD (early bird) or 75$ CAD. Once enrolled, participants received a link via email and the training module was available from the 20 ^th^ of March to the 31 ^st^ of March 2017. The team decided to place a time limit for module completion to stimulate participants to complete the training. The access to the online module was asynchronous; participants could complete the vignettes at their convenience. The entire module required approximately 2 hours to complete and it was possible to pause the training, review past questions and take-it up at a different time.

**Figure 1.**
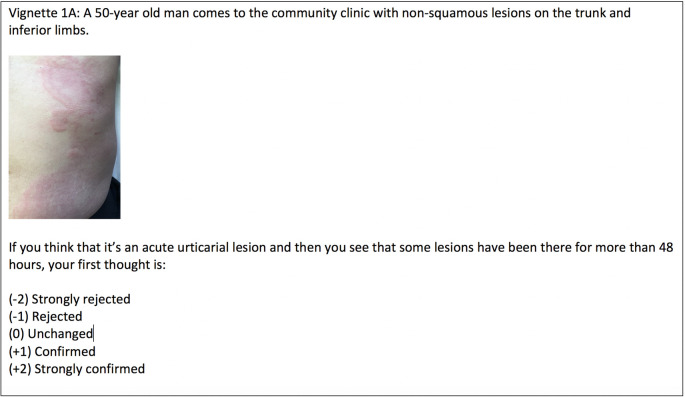
Vignette 1A on acute urticaria

Each vignette addresses a specific topic and starts with an initial hypothesis about the lesions seen on the photograph. In each question, a new element of the clinical situation is presented and the participant must assess the impact that the new information has on their initial hypothesis. The questions are aimed to make participants reflect about the key dermatologic aspects associated with the selected lesion. Participant responses are given on a 5 point Likert scale: strongly negative impact (-2), negative impact (-1), no impact act all (0), positive impact (+1) and strongly positive impact (+2). The total number of questions for module 1 (8 vignettes) was 77. Table 1 shows the distribution and topics of the vignettes. Figure 1 presents a sample clinical vignette (vignette 1A).

In order to support acquisition of basic concepts related pathologies presented in this module, each vignette was followed by a short training capsule (narrated power point presentation) that lasted a few minutes. These capsules, in PDF format without the pictures, were available for download online for some time after the training.

At the end of each vignette (after the short training capsule), a pop-up window appeared inviting the participants to write outstanding questions about which they would like to receive greater details from the experts. The outstanding questions were addressed during a 1-hour telephone conference held on April 6 ^th^, a week after the group completed all the vignettes. Sample outstanding questions are presented in Table 1.

### Dermatology Expert Feedback

**Figure 2.**
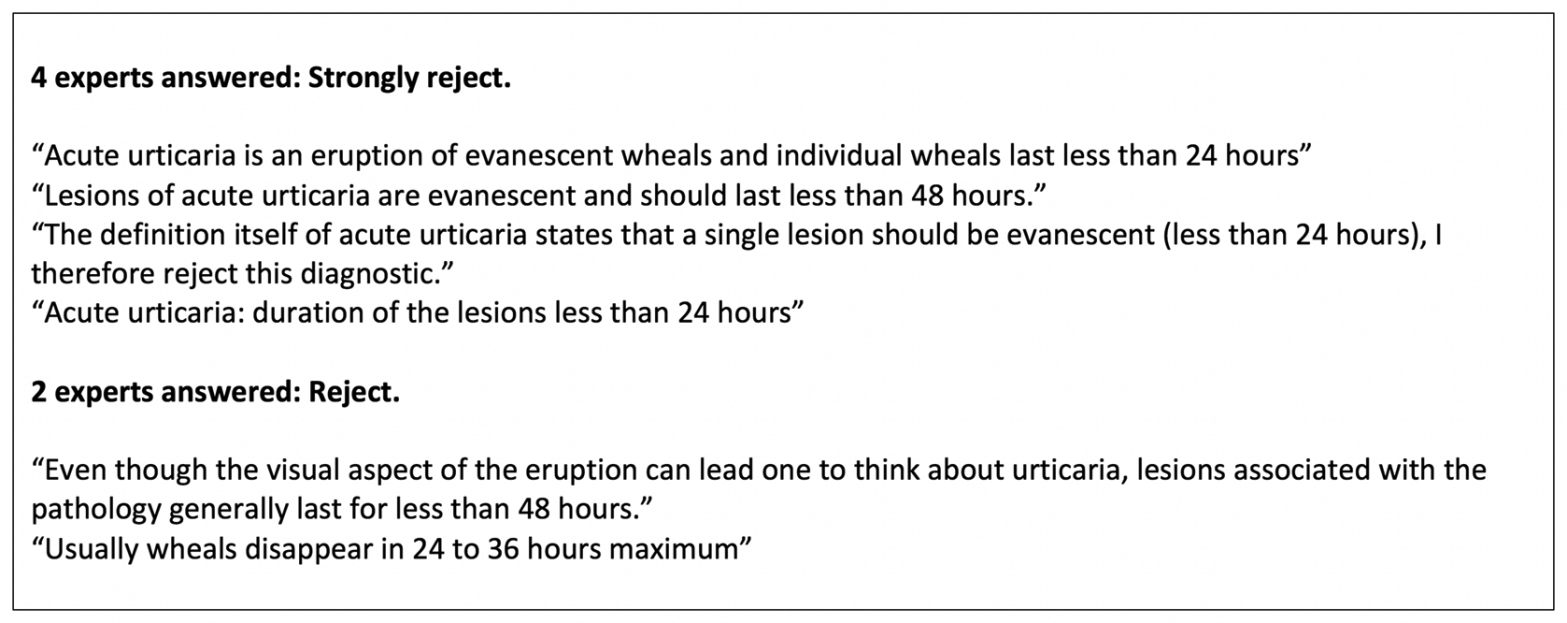
Example of expert feedback (for vignette 1A)

Four dermatologists and two senior residents in dermatology served as experts. They completed the 8 clinical vignettes, by clicking one of the choices on the Likert scale and giving brief justifications for their answers. These answers and justifications were deposited on the online platform so that participants could access them immediately after having answered each question. Figure 2 presents an example of expert feedback about vignette 1A in Figure 1. It is important to note that expert answers and justification were not identical. This aspect of LbC is crucial as it introduces the possibility that more than one answer can be correct. ( Foucault et al. 2014)

### Assessment of LbC Experience

A total of 45 family physicians took part in the initial LbC-CPD experience. At the end of the session, (N=45) participants were invited to respond to a satisfaction questionnaire (see Appendix for survey questions), where they could leave comments. The questions were appended to the last vignette, so participants would respond immediately after completing the module. This explains the 98% response rate (n=44) to this questionnaire.

A 1-hour telephone conference to address the outstanding questions was attended by 30 physicians. A survey about the effectiveness of this phone conference was also conducted. The response to this survey was 36%. Immediately, following the telephone conference, a 30-minute focus group was held with five participants that volunteered to remain on-line. The purpose was to enquire about the global experience.

## Results

**Figure 3.**
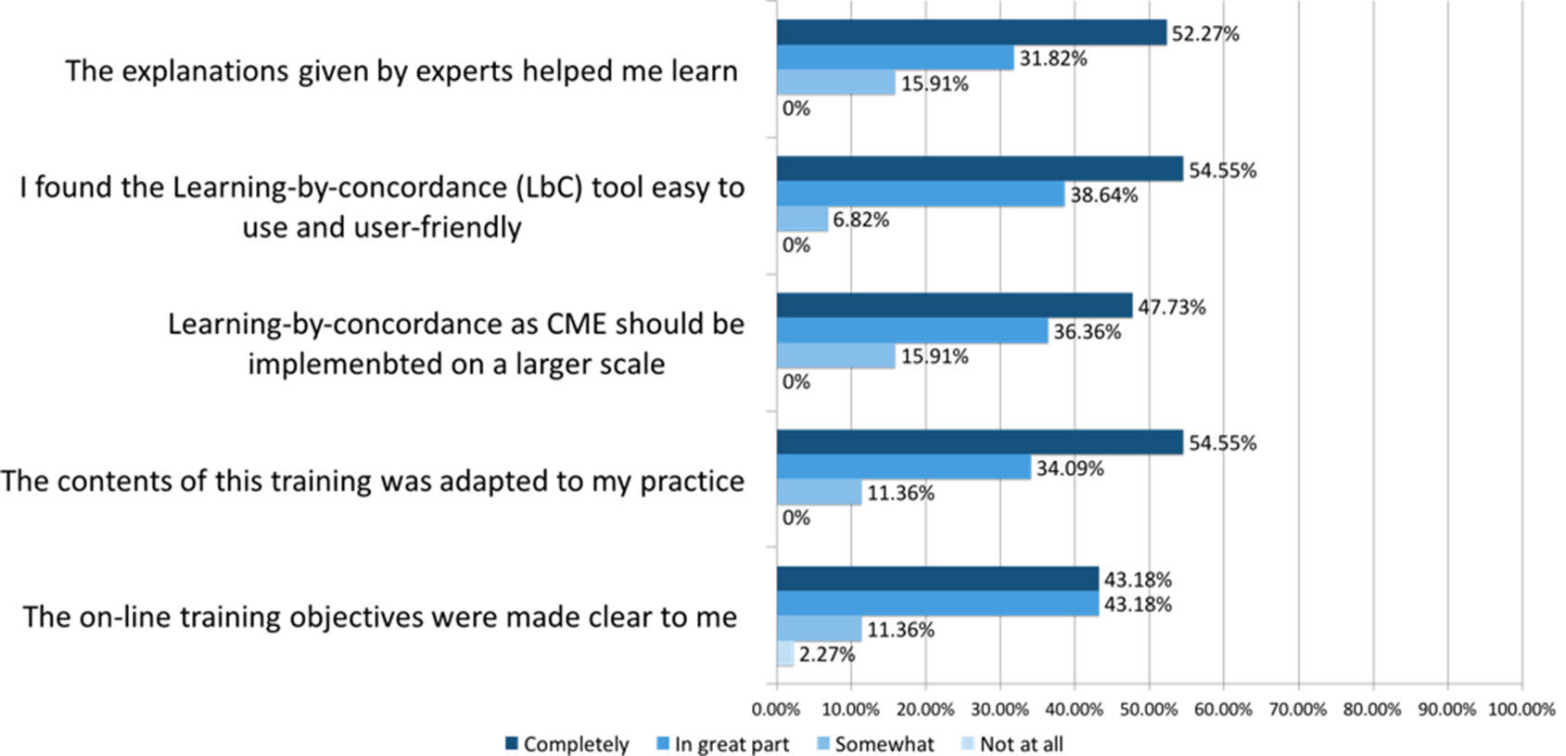
Survey Results CME=Continuous Medical Education

Figure 3 presents some of the survey results (satisfaction questionnaire). They show that the objectives of the training module were clear and that the material in the programme allowed participants to increase their knowledge (87%). Survey results indicate that instructor explanations were deemed useful for learning by respondents. Participants also found that the contents of the training were adapted to family physician practice and that it was useful for improving their knowledge and interventions in dermatology cases (87%). In general, most participants found the online training tool user friendly and indicated that LbC as CPD should be implemented in a larger scale (i.e. more LbC - CPD training).

As for the conditions for completing the training, 84% of respondents used a desktop computer, 11% a tablet and 5% used a smartphone. As for time of day, 13 participants completed the training in the morning, 15 in the afternoon and 17 in the evening. Seven participants completed the training while they were outside the province of Quebec. Most (81%), found that the length of the training was just right; surprisingly, 19% found it too short.

Comments shared in the focus group indicated that this training was more efficient, interesting and stimulating than reading a dermatology manual on your own. In general, they indicated that they appreciated the format and the freedom it afforded. Participants found the narrated capsules useful and stimulating. They also liked to be able to think their responses through before submitting them. Having access to expert’s responses and justification that varied slightly was very interesting. It became clear that the tool allowed participants to improve their clinical reasoning and was easy to fit into a busy schedule. Finally, participants appreciated having access to some of the materials (narrated capsules) after the training was completed.

Appreciation of the teleconference was not high. Participants found issue with the format, mentioning that although their questions were answered, it was difficult to follow the instructor’s answers and explanations. They mentioned that there was too much information for the short time span which made it difficult to follow without taking notes. Some mentioned that a written document with the outstanding questions and brief answers would have been helpful. Technical difficulties were also mentioned, the volume on the phone conference was not high enough and the quality of the connection was not very good. This probably prevented rich interaction between the expert and the participants.

## Discussion

In-service family physicians are immersed in clinical work, especially in our province where government policies are putting pressure on them to increase their patient case-load. This raises the importance of CPD and therefore innovative solutions must be sought to enhance the experience and achieve appropriate learning goals. The results of our current study indicate that in-service physicians found the tool user-friendly and allowed them to acquire useful knowledge for their practice.

DPC is evolving to produce training modules that allow professionals themselves to identify their knowledge gaps and determine when they need to update their skills. Because LbC vignettes are cases that are familiar to practicing physicians, challenging their initial hypothesis makes them become aware of their reasoning process and effectively identify their knowledge gaps. Furthermore, by having access to expert explanations, participants learn to discriminate the important aspects from the unimportant aspects and ultimately make better clinical decisions. Furthermore, including quality educational material immediately after each vignette serves to reinforce basic concepts of dermatology. The outstanding questions that can be asked in the pop-up window allow participants to review concepts they have not understood properly. Finally, the phone conference serves to consolidate the new learning.

We also considered it important to limit the time for the training to be completed to 14 days. Knowing that after this period, they would no longer get access to the module, “forced” participants to complete the training. Their engagement was also enhanced by the fee for accessing the training. Finally, the targeted phone conference to address outstanding questions also served to maintain interest and engagement.

We consider this teleconference as an important component of the CPD LbC training experience. It provides the opportunity for participants to interact with the instructor and get answers to their questions. Beside the technical difficulties we experienced, we are aware that the format needs to be reviewed. The instructor felt awkward delivering a speech into the phone without knowing if and how people were listening. The format needs to incorporate a visual component (written document, powerpoint presentation, videoconferencing) and a way needs to be found to encourage participants to ask questions directly to the instructor.

## Conclusion

This study provides further evidence of the relevance of learning by concordance for the specific context of continuing medical education provided to a geographically dispersed population. The principal strengths of LbC are that the clinical situations around which the learning task revolves are authentic and reflect typical tasks of a primary physician’s daily practice. Also, as participants verify immediately the concordance of their answers with those of experts in the field, and think about the differences, learning is supported more than if the participants simply read up on the subject or attended a lecture. Finally, the LbC can be completed at anytime and anywhere, hence relieves many of the constraints that typically haunt CPD planners.

The study revealed that the LbC modality is effective. Instead of presenting the basic material organized in a linear text-book table of contents list of topics, the tool presented clinical vignettes and images of lesions that participants might have encountered in their practice. It is expected that introducing cases in such a way, with a view of activating prior knowledge and experience, a basic learning principle according to cognitive science, helps to better embed new knowledge in the specialized scripts within semantic memory. Results indicating that participants recognized that they effectively acquired new knowledge through the tool and that this knowledge was useful for them in their practice reinforce the claim the LbC constitutes an efficient vehicle for acquiring situated knowledge.

## Take Home Messages

• A persistent challenge for Continuous Professional Development (CPD) planners is providing solutions for practicing clinicians to help them juggle clinical work, personal life and in-service training.
• The Learning-by-concordance (LbC) online tool can be completed anytime and anywhere.
• The strength of LbC is that the clinical situation around which the learning task revolves is an authentic part of a primary physician’s practice.
• As participants confront their answers to those of experts in the field, and think about the differences, learning is supported more than if the participants simply read up on the subject or attend a lecture.

## Notes On Contributors

***Julie Lecours***, MD, is a senior resident in dermatology at Université de Montréal. Prior her medical studies, she worked a few years as a physical therapist and she completed a Master’s degree in Biomedical Sciences at Université de Montréal.

***Fanny Bernier***, MD, just finished her 5 years residency in dermatology at Université de Montréal. She is now working full time as a dermatologist in Montreal and surroundings.

***Dominique Friedmann***, MD, is a dermatologist at Centre hospitalier de l’Université de Montréal (CHUM). She completed a specialization in autoimmune bullous diseases, severe cutaneous drug eruption and cutaneous lymphoma in Paris, France.

***Vincent Jobin***, MD is a pulmonologist and is the head of the CPD office at the Faculty of Medicine of the Université de Montréal.

***Bernard Charlin***, MD, PhD, is a Professor at the Department of Surgery at Université de Montréal. He holds a Master’s degree in Education from Harvard University and a PhD in Education from the University of Maastricht. His research field is reasoning in the context of uncertainty (theory, acquisition, assessment).

***Nicolas Fernandez***, PhD, is a Professor at the Department of Family Medicine and Emergency Medicine at Université de Montréal. He holds a PhD - Education, in Higher Education. He is the director of a Master’s program in pedagogy for Health Educators and conducts research in the field of Health Sciences Education. https://orcid.org/0000-0002-3840-2641

## Appendices

APPENDIX

Survey questions (Satisfaction questionnaire)

Please select the degree to which you agree with the following statements

Strongly disagree, disagree, indifferent, agree, strongly agree

• The on-line training objectives were clearly communicated to me.
• The content material of the on-line training was adapted to my practice
• Learning-by-concordance as a CPD training tool should be implemented in a larger scale
• I found the Learning-by-concordance tool easy to use and user-friendly.
• The expert explanations helped me learn
• My interest for this type of training is: strong, medium, low.
• The length of the training was: too long, just right, too short
• I completed the LbC training tool on my: tablet, smartphone, desktop computer
• The content material allowed me to increase my knowledge

Comments or suggestions
