# Supplementary material for: Learning-by-Concordance for Family Physicians: Revealing its Value for Continuing Professional Development in Dermatology
Source: MedEdPublish (2016). 2018 Oct 17;7:236. [Version 1] doi: 10.15694/mep.2018.0000236.1 (PMC10712009; doi:10.15694/mep.2018.0000236.1)
Supplement: Peer review history

# Reviewer report: Reviewer response for version 1

**DOI:** 10.21956/mep.19695.r29446

**Published:** 2019-06-03

**Authors:** Ken Masters

**Affiliations:** 1 Sultan Qaboos University

This review has been migrated. The reviewer awarded 3 stars out of 5 An interesting paper using learning-by-concordance methods for family physicians taking a CPD online course in Dermatology. The authors establish the situation clearly, although, perhaps, they could have gone into more detail (more than a single line) explaining the need for Primary Care physicians to brush up on their skills in this area. In addition, although script concordance methodologies for teaching and assessment are becoming more popular, they are still not widely known or used, so a more detailed explanation probably would have helped most readers understand the way the systems work.The use of a pilot before implementation (a step frequently overlooked by researchers) added robustness to the project. The strict time limit of the course is somewhat unusual, but, given that the participants knew about it before-hand, they would have been able to make appropriate arrangements. The follow-up questions and telephone sessions (apart from the reported technical problems) may make the course difficult to implement on a wider scale (perhaps online forums would be better), but, for a small number of participants, appears to have been reasonably workable.Two issues with the paper:• The paper refers to Table 1. Unfortunately, Table 1 appears to have been omitted from the paper. This is most unfortunate, as, from the description of Table 1 in the text, it would give an overview of the course as a whole.• The Discussion really should relate the work back to the literature, but this has not been done in a meaningful way. Although this is not a full-blown research article, some more reference to the literature (in both the Introduction and the Discussion) would help to position the paper more clearlySo, an interesting development, and the researchers appear to have created something that will make a useful contribution to CPD Dermatology. The absence of the Table and lack of literature, though, does make the paper less valuable than it might have been.

Reviewer Expertise:

NA

No decision status is available

# Reviewer report: Reviewer response for version 1

**DOI:** 10.21956/mep.19695.r29445

**Published:** 2018-10-18

**Authors:** Leila Niemi-Murola

**Affiliations:** 1 University of Helsinki

This review has been migrated. The reviewer awarded 4 stars out of 5 Thank you for the invitation to review this interesting article. Technology-enhanced learning is a hot topic and this kind of intervention is very feasible for dermatology, the diagnostic process based on visual material. Technology-enhanced learning modules are often based on the latest technological tricks and I greatly appreciated the fact that the authors have used educational theories as their starting point. The authors have piloted the tool with a small number of participants and an equal number of expert viewers had a chance to review the tool. Forty-five primary care physicians were the target group of this educational intervention and the results show that they were very satisfied with the learning experience.Electronic format allows the authors also to collect feedback from the learners in order to fine tune the intervention and make it even more learner-friendly. The authors share the critical comments with the readers, which is very valuable for those planning similar educational interventions.

Reviewer Expertise:

NA

No decision status is available
